# Comparative toxicity and biochemical impacts of certain recommended insecticides against *Spodoptera littoralis* (Lepidoptera: Noctuidae)

**DOI:** 10.1038/s41598-026-48788-6

**Published:** 2026-04-28

**Authors:** A. E. El-morshedy, A. A. M. Shalaby, H. M. H. Al-Shannaf, Mohammed G. Mahmoud, Hesham A. M. Ibrahim, M. Y. Hendawi

**Affiliations:** 1 Department of Cotton Leafworm, Plant Protection Research Institute, Agricultural Research Center, Dokki, 12622 Giza Egypt; 2 Plant Protection Department, Faculty of Agriculture, Zagazig University, Zagazig, Egypt; 3 Department of Agricultural Zoology and Nematology, Faculty of Agriculture, Assiut Branch, Al Azhar University, Assiut, 71524 Egypt

**Keywords:** Chlorantraniliprole, Indoxacarb, Lufenuron, Emamectin benzoate, *Spodoptera littoralis*, Biochemical technologies, Biochemistry, Chemical biology, Ecology, Ecology, Environmental sciences, Zoology

## Abstract

The cotton leafworm, *Spodoptera littoralis* (Lepidoptera: Noctuidae), is a destructive agricultural pest that causes significant yield losses in various crops. The extensive use of chemical insecticides necessitates a comprehensive evaluation of their toxicological efficacy and sublethal biochemical effects to support sustainable pest management strategies. This study aimed to evaluate the comparative toxicity and biochemical effects of four insecticides (emamectin benzoate, chlorantraniliprole, lufenuron, and indoxacarb) against fourth-instar larvae of laboratory-reared cotton leafworm *Spodoptera littoralis* (Boisd.). Among all the tested insecticides, emamectin benzoate was the most effective (LC_50_= 0.399 mg/L), whereas indoxacarb was the least effective (LC_50_= 13.062 mg/L). Sublethal effects were assessed by examining the biochemical responses of fourth-instar larvae exposed to LC_25_ concentrations of 0.19, 3.38, 3.052, and 8.008 mg/L for the respective insecticides under controlled laboratory conditions. Enzymatic activity was monitored at 1, 3, and 5 days post-treatment. The results revealed significant alterations in total protein and carbohydrate contents, as well as the activities of key enzymes involved in carbohydrate metabolism (invertase, trehalase, and amylase), transamination (AST and ALT), phosphatase function (ALP), and detoxification enzymes (α-esterase), compared with those in the untreated controls. These findings provide mechanistic insights into insecticide-induced metabolic and enzymatic disruptions and highlight the importance of incorporating biochemical indicators into pesticide evaluation frameworks to support more effective and sustainable insect pest management programs.

## Introduction

Cotton (*Gossypium hirsutum* L.) is considered one of the most economically significant crops in Egypt and globally, as it is cultivated in more than 100 countries^1^. The cotton leafworm (*Spodoptera littoralis* (Boisduval) (Lepidoptera: Noctuidae)) is a highly destructive and polyphagous insect pest that infests a wide variety of field crops across different regions, including tropical and subtropical zones^2^. *S. littoralis* is known to feed on approximately 90 economically important plant species across 40 botanical families^3^. The extensive application of insecticides for controlling *S. littoralis* has contributed to the development of resistance against numerous registered chemical agents^4^. Resistance to insecticides remains a persistent and growing challenge in arthropod pest management, necessitating the introduction of novel insecticides such as chlorantraniliprole (CAP). Chlorantraniliprole exhibits strong efficacy against chewing pests through both ingestion and contact modes of action. This anthranilic diamide insecticide binds to ryanodine receptors in insect muscle cells, causing unregulated release of calcium ions from internal stores within the sarcoplasmic reticulum, which ultimately results in feeding cessation, paralysis, and insect death within 24–72 h^5–7^. Emamectin benzoate represents a key member of the avermectin class of insecticides. Avermectins, including emamectin benzoate, have demonstrated high efficacy against a broad spectrum of arthropod pests^8^. Emamectin benzoate functions as a chloride channel activator, leading to decreased neuronal excitability. According to Grafton-Cardwell et al.^9^., insect larvae exposed to emamectin benzoate quickly cease feeding, become irreversibly paralyzed, and die within 3 to 4 days. Lufenuron is a nonconventional insecticide belonging to the benzoylurea chemical class. During molting and growth, lufenuron disrupts the proper synthesis of chitin, an essential component of the insect cuticle. Insect growth regulators (IGRs) are classified as third-generation insecticides because of their unique mode of action, which differs from conventional insecticides and offers advantages such as low toxicity to beneficial organisms and minimal environmental contamination.

The high effectiveness of certain insect growth regulators (IGRs) provides a promising alternative approach for managing *S. littoralis*, particularly in light of the resistance that has emerged against conventional insecticides^10^. Indoxacarb is an insecticide characterized by a unique mode of action and proven efficacy against a wide range of insect pests. It is classified within the oxadiazine chemical group. Insects are exposed to indoxacarb primarily through the ingestion of treated plant material or bait formulations, and indoxacarb may also penetrate through direct contact with the cuticle. Following entry into the insect body, specific metabolic enzymes, mainly esterases or amidases, located in the gut and fat tissues convert indoxacarb into its highly potent metabolite, known as N-demethoxycarbonyl indoxacarb (DCJW)^11^. This active metabolite (DCJW) irreversibly binds to voltage-gated sodium channels in the nervous system of insects. Voltage-gated sodium channels are essential for the propagation of nerve impulses. Blocking these channels disrupts the normal movement of sodium ions, resulting in cessation of feeding, impaired locomotion and induced tremors, paralysis, and ultimately death. Insecticides from distinct chemical classes, including chlorantraniliprole, emamectin benzoate, lufenuron, and indoxacarb, were selected for comparative investigation. This study aimed to evaluate their toxicological impacts on fourth-instar larvae of *S. littoralis*, in addition to assessing their influence on specific biochemical processes through the modulation of key enzymatic activities.

## Materials and methods

### Toxicity study

#### Test insects

In accordance with El-Defrawi^12^, a laboratory strain of *S. littoralis* was acquired from the Central Laboratory of Pesticides, Agricultural Research Center (ARC) in Cairo, Egypt, and reared on castor oil leaves for many years under laboratory conditions (temperature of 26 ± 2 °C and relative humidity 65 ± 5%). The fourth-instar larvae were selected as a representative mid-larval stage due to their active feeding and high metabolic and detoxification activities, making them suitable for toxicological and biochemical assessments. Earlier instars are more sensitive, while later instars may undergo physiological changes related to pupation that can affect enzyme activity. This stage is widely used in studies on *Spodoptera littoralis*, supporting its reliability for such investigations^13–18^.

### Test pesticides

Four insecticides were used in this study: chlorantraniliprole (Coragen 20% SC), emamectin benzoate (Speedo 5.7% WG), indoxacarb (Strong 30% SC) and lufenuron (Sunuron 5% EC). The formulation type, mode of action and supplier details are highlighted in Table 1.

**Table 1:** Information about the insecticides tested against 4^th^ instar of *S. littoralis*.

Active ingredients	Formulation	Chemical Group	IRAC MoA Group	Manufacturer
**Chlorantraniliprole**	20% SC	28 A, Diamides	Diamide, ryanodine receptor modulator	Sun-Dot (S) PTE Ltd.
**Emamectin benzoate**	5.7% WG	6 A, Avermectins	Avermectin, glutamate gated chloride channel allosteric modulator	Dakang Jifini Biochemical Ltd.
**Indoxacarb**	30% SC	22 A, Oxadiazines	Oxadiazine, voltage gated chloride channel blocker	Jiangsu Subin Agrochem Co., Ltd.
**Lufenuron**	5% EC	15, IGRs	IGRs, inhibit the enzyme chitin synthase 1 (CHS1)	Sun-Dot (S) PTE Ltd.

SC: suspension concentrate; WG: water-soluble granule; EC: emulsifiable concentrate.

### Toxicity bioassay

The leaf dipping approach was used to evaluate the larvicidal effects of the tested insecticides on one day-old 4^th^ instar larvae. Fresh castor bean leaf disks, each with a diameter of 3 cm, were prepared via a cork borer. Five concentrations of each insecticide were prepared in distilled water by serial dilution from a stock solution, i.e., 20, 15, 10, 5 and 2 mg /L for chlorantraniliprole; 1, 0.7, 0.5, 0.3 and 0.1 mg/L for emamectin benzoate; 25, 20, 15, 10 and 5 mg/L for indoxacarb and 20, 15, 10, 5 and 2 mg/L for lufenuron. These disks were then individually immersed in the predetermined test concentrations for 15 s. Following immersion, the treated disks were allowed to air dry under ambient conditions. A single larva was subsequently introduced to each treated leaf disk within individual plastic cylinder tubes (3.4 cm diameter, 7.0 cm height). Larvae were permitted to feed on the treated disks for 24 h; afterwards, they were fed fresh and untreated castor leaves until the end of the experimental period. To ensure adequate aeration, each tube was fitted with a perforated plastic lid. Excess moisture within the tubes was mitigated by the placement of filter paper at the base. The experimental design included three replicates for each concentration, with each replicate comprising 10 larvae, resulting in a total of 30 larvae per concentration. Control groups were established using leaf disks dipped in distilled water to account for baseline mortality. The bioassay was conducted in a locally manufactured incubator equipped with a digital temperature controller to maintain a constant environment. The larvae were kept at 26 ± 2 °C with a relative humidity of approximately 65 ± 5%. A photoperiod of 12:12 h (light: dark) was provided via standard LED lighting. Larval mortality was assessed by observing the absence of movement in response to a gentle brush.

### LC value determination

The selected concentration ranges were based on previously reported LC₅₀ values of the tested insecticides against *Spodoptera littoralis*, ensuring that the tested doses encompassed a suitable mortality range (20–80%) required for accurate probit analysis. This approach is consistent with earlier toxicological studies, which recommend selecting concentrations that bracket expected LC₅₀ values to obtain reliable and reproducible estimates^19–22^. A total of 150 larvae were used in each treatment (10 larvae / 3 replicates / 5 concentrations of each compound). The LC_25_, LC_50_ and LC_90_ values were calculated according to Finney^23^. Sun’s equation^24^ was used to calculate the toxicity index (T.I.) as follows: Toxicity index = LC_50_ or LC_90_ of the most efficient compound / LC_50_ or LC_90_ of the other compound × 100. The relative potency (RP) was determined using the method of Zidan and Abdel-Megeed^25^ as follows: relative potency (fold) = LC_50_ or LC_90_ of the lowest efficient compound / LC_50_ or LC_90_ of the other compound.

### Biochemical assay

In a separate experiment, the LC_25_ of each compound was applied to fourth-instar larvae to investigate the resulting biochemical alterations. This investigation aimed to elucidate the extent of these effects and to ascertain variations in their magnitude attributable to the distinct chemical classes of the pesticide treatments. The experiment was conducted in triplicate for each treatment. Surviving fourth-instar larvae were collected at 1, 3 and 5 days post-treatment to determine total protein, total carbohydrate, carbohydrate-hydrolyzing enzymes, aspartate aminotransferase (AST), alanine aminotransferase (ALT), alpha esterase and alkaline phosphatase (ALP) levels; untreated larvae were used as controls.

### Chemicals

The bovine albumin standard was purchased from Stanbio Laboratory (Texas, USA). Coomassie Brilliant Blue G-250 was obtained from Sigma (Sigma Chemical Co.). P-Nitroanisole (purity 97%) was obtained from Ubichem Ltd. (Hampshire), whereas nicotinamide adenine dinucleotide phosphate (reduced form, NADPH) was obtained from BDH Chemicals Ltd. (Poole, England). The remaining chemicals were of high quality and purchased from commercial local companies.

### Apparatus

Larvae were homogenized for biochemical analysis in a chilled glass Teflon tissue homogenizer (ST-2 Mechanic-Preczyina, Poland). After homogenization, the supernatants were stored in a deep freezer at -20 °C for a short period, not exceeding 48 h until use for biochemical assays. A double beam ultraviolet/visible spectrophotometer (Spectronic 1201, Milton Roy Co., USA) was used to measure the absorbance of colored substances or metabolic compounds.

### Preparation of insects for analysis

The insects were prepared as described by Amin^26^. The samples were homogenized in distilled water (50 mg/ml) as the homogenization medium only for total protein and carbohydrate determinations, where enzyme preservation was not required. For enzymatic activity assays, samples were homogenized in 0.1 M phosphate buffer (pH 7.0) to maintain pH stability and preserve enzyme integrity according to established protocol. The homogenates were centrifuged at 8000 r.p.m. for 15 min at 2 °C in a refrigerated centrifuge. The deposits were discarded and the supernatants, which are referred to as enzyme extracts, could be stored for at least one week without appreciable loss of activity when stored below 0 °C.

### Colorimetric determination

The total protein content in the supernatants of the homogenate-treated larvae of *S. littoralis* was determined as described by Bradford^27^. Total carbohydrates were extracted and prepared for the assay according to the method of Crompton and Birt^28^ and were estimated in the acid extract of sample via the phenol-sulphuric acid reaction of Dubois et al.^29^. Aspartate aminotransferase (AST; EC 2.6.1.1) and alanine aminotransferase (ALT; EC 2.6.1.2) levels were determined colorimetrically according to the methods of Reitman and Frankle^30^. Trehalase (EC 3.2.1.28), amylase (EC 3.2.1.1) and invertase (EC 3.2.1.26) enzymes were determined similarly to those described by Ishaaya and Swiriski^31^. Alpha esterase (α-esterase) activity was determined according to Van Asperen^32^ using α-naphthyl acetate as a substrate. Alkaline phosphatase (ALP; EC 3.1.3.1) was accomplished by using P-nitrophenyl phosphate as a reaction substrate^33^. All biochemical and enzymatic assays were performed according to their respective standardized protocols under optimized experimental conditions specific for each enzyme (temperature, incubation time, reaction volume, and buffer system). Appropriate reagent blanks and untreated controls were included in all assays to correct for background absorbance and ensure valid comparative analysis. All determinations were carried out in triplicate. The percentage changes in total protein and total carbohydrate contents were calculated to determine the differential effects of the treatments relative to those of the untreated controls as follows:

$$\:\mathrm{C}\mathrm{h}\mathrm{a}\mathrm{n}\mathrm{g}\mathrm{e}\:\left(\mathrm{C}\:{\%}\right)=\:\frac{\mathrm{T}\mathrm{r}\mathrm{e}\mathrm{a}\mathrm{t}\mathrm{m}\mathrm{e}\mathrm{n}\mathrm{t}-\mathrm{C}\mathrm{o}\mathrm{n}\mathrm{t}\mathrm{r}\mathrm{o}\mathrm{l}}{\mathrm{C}\mathrm{o}\mathrm{n}\mathrm{t}\mathrm{r}\mathrm{o}\mathrm{l}}\times\:100$$

The impact of each treatment on enzymatic activity was assessed by calculating the percentage change in the activity of each enzyme relative to that of the untreated control as follows:

$$\:\mathrm{R}\mathrm{e}\mathrm{l}\mathrm{a}\mathrm{t}\mathrm{i}\mathrm{v}\mathrm{e}\:\mathrm{a}\mathrm{c}\mathrm{t}\mathrm{i}\mathrm{v}\mathrm{i}\mathrm{t}\mathrm{y}\:\left(\mathrm{R}\mathrm{A}\:{\%}\right)=\:\frac{\mathrm{T}\mathrm{r}\mathrm{e}\mathrm{a}\mathrm{t}\mathrm{m}\mathrm{e}\mathrm{n}\mathrm{t}-\mathrm{C}\mathrm{o}\mathrm{n}\mathrm{t}\mathrm{r}\mathrm{o}\mathrm{l}}{\mathrm{C}\mathrm{o}\mathrm{n}\mathrm{t}\mathrm{r}\mathrm{o}\mathrm{l}}\times\:100$$

### Statistical analysis

The obtained results were statistically analyzed by one-way analysis of variance (ANOVA) according to Tukey’s HSD^34^. The data were subjected to statistical analyses using the software package Costat^®^ Statistical Software^35^.

## Results

### Comparative toxicity of the tested insecticides to 4^th^ instar larvae of *S. littoralis*

The toxicity index was calculated by evaluating the relative efficacy of the tested insecticidal compounds at specific concentrations in comparison with a highly toxic reference compound. Emamectin benzoate was designated as the standard compound for determining the toxicity index, and its value was fixed at 100 in all cases. Potency levels are expressed as fold differences and were calculated by dividing the LC_50_ or LC_90_ values of indoxacarb (selected as the low-toxicity reference compound) by the corresponding values of each tested insecticide. Emamectin benzoate exhibited the highest toxicity (LC_50_ = 0.39 mg/L) and showed more than 30-fold greater potency than did indoxacarb (LC_50_ = 13.06 mg/L), making it the most effective compound tested. At both the LC_50_ and the LC_90_, emamectin benzoate demonstrated the highest potency among all the evaluated insecticides. The toxic effects of all the treatments were assessed at 72 h post-application. The ranking of insecticide efficacy against fourth instar larvae varied between the LC_50_ and LC_90_ levels. At the LC_50_, indoxacarb had the lowest efficacy, whereas at the LC_90_, chlorantraniliprole was the least effective (Table 2). The slopes of the regression lines indicated relatively uniform susceptibility of *S. littoralis* larvae to the tested compounds. On the basis of the estimated LC_50_ and LC_90_ values, fourth-instar larvae exhibited moderate susceptibility to all the evaluated insecticides.

**Table 2:** Toxicity of emamectin benzoate, chlorantraniliprole, lufenuron and indoxacarb to 4^th^ instar larvae of *S. littoralis*.

Insecticides	LC_25_	LC_50_ (mg/L)	LC_90_ (mg/L)	Slope ± SE	Toxicity index at		Relative potency fold at		LC_90_/LC_50_
	(mg/L)				LC_50_	LC_90_	LC_50_	LC_90_	
Emamectin benzoate	0.190	0.399	1.648	0.864 ± 0.090	100.00	100.00	32.70	20.09	4.13
Chlorantraniliprole	3.380	9.995	78.521	1.0 ± 2.828	4.00	2.10	1.31	0.42	7.86
Lufenuron	3.052	8.479	59.153	0.928 ± 1.528	4.71	2.79	1.54	0.56	6.98
Indoxacarb	8.008	13.062	33.112	0.978 ± 1.648	3.06	4.98	1.00	1.00	2.53

Toxicity index and relative potency based on the LC_50_.

### Biochemical responses

The present study explored the physiological responses of *S. littoralis* larvae to four recommended insecticides. A range of metabolic and enzymatic markers were assessed at 1, 3 and 5 days post-treatment. The findings demonstrate differential impacts depending on the mode of action of each insecticide.

### Total protein

**Fig. 1:**
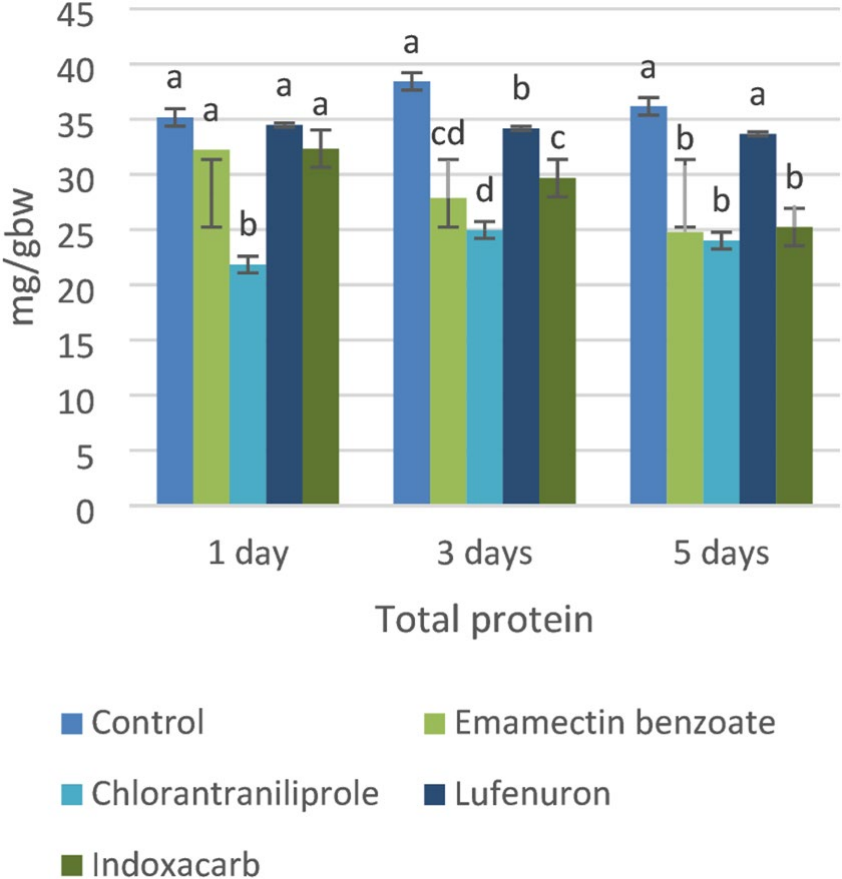
Biochemical changes in 4^th^instar larvae of S. *littoralis* treated with the LC_25_ of the tested insecticides: total protein and total carbohydrates. The data are expressed as the means ± standard deviations (STDEVs). The values given are the means of three replicates. The values for each day marked by the same letters are not significantly different according to with Tukey’s HSD test at *p* < 0.05. Those with different letters are significantly different.

The total protein levels in the control group remained relatively stable throughout the experimental period, ranging from 35.17 to 38.43 mg/g.b.w., indicating normal metabolic conditions. Treatment with chlorantraniliprole and indoxacarb resulted in progressive and significant reductions in total protein content. By day 5, the protein levels had declined to 24.00 and 25.23 mg/g.b.w., respectively. Similarly, emamectin benzoate caused a marked decrease in total protein levels, from 32.23 to 24.77 mg/g.b.w. over the exposure period. In contrast, lufenuron had a minimal effect on the total protein content, maintaining values close to those of the control group throughout the experimental duration (Fig. 1).

### Total carbohydrates

The carbohydrate levels in the control larvae moderately fluctuated throughout the experimental period, ranging from 33.23 to 36.07 mg/g.b.w., indicating normal metabolic activity. Chlorantraniliprole and indoxacarb produced the most pronounced reductions in carbohydrate content. By day 5, carbohydrate levels had decreased to 8.17 and 6.43 mg/g.b.w., respectively. Emamectin benzoate also significantly reduced the carbohydrate content, with values ranging between 13.57 and 14.63 mg/g.b.w. during the exposure period. In contrast, lufenuron maintained relatively high carbohydrate levels (29.46–30.93 mg/g.b.w.), remaining close to those of the control group (Fig.1).

### Carbohydrate-hydrolyzing enzymes

The data shown in Table 3 were used to evaluate the impact of the tested insecticides on the activity of key carbohydrate-metabolizing enzymes (invertase, trehalase, and amylase) in *S. littoralis* larvae over three time intervals post-treatment (1, 3, and 5 days). The results provide valuable insight into how these insecticides interfere with energy metabolism and larval survival through enzymatic disruption. The inhibition of carbohydrate enzymes particularly trehalase and amylase, by chlorantraniliprole and indoxacarb severely disrupts energy homeostasis in *S. littoralis*. Emamectin benzoate had intermediate effects, whereas lufenuron was the least metabolically disruptive. These trends are consistent with earlier observations of reduced total carbohydrate content under the same treatments.

### Invertase activity

Invertase activity in the control group remained high and stable throughout the experimental period. Chlorantraniliprole treatment resulted in the most pronounced reduction in invertase activity, which progressively decreased from 59.9 µg glucose/min/g.b.w on day 1 to 38.8 µg glucose/min/g.b.w on day 5. Compared with the control, indoxacarb also caused a significant decrease in invertase activity. Emamectin benzoate induced a moderate reduction in invertase levels, with a slight recovery observed over time. In contrast, lufenuron maintained invertase activity at levels comparable to those of the control across all the sampling intervals (Table 3).

**Table 3:** Changes in carbohydrate enzyme activities (µg glucose/min /g.b.w) in the 4^th^ larval instars of *S. littoralis* treated with the LC_25s_ of the tested compounds.

Treatment		Invertase			Trehalase			Amylase		
		1 day	3 days	5 days	1 day	3 days	5 days	1 day	3 days	5 days
Control	Conc.*	84.733ab ± 1.950	96.533a ± 1.305	91.767ab ± 2.060	58.833a ± 1.258	85.267a ± 2.969	64.467a ± 0.473	78.633a ± 2.203	96.767a ± 2.108	89.933a ± 1.601
Emamectin benzoate	SA**	67.533c ± 2.403	78.833b ± 2.196	88.267b ± 2.354	18.067b ± 2.001	18.367c ± 0.723	21.767b ± 1.861	21.833c ± 1.607	19.833c ± 2.098	29.967b ± 2.550
	RA%***	-20.30	-18.36	-3.81	-69.29	-78.46	-66.24	-72.23	-79.50	-66.68
Chlorantraniliprole	SA	59.867c ± 2.303	35.567d ± 1.172	38.833d ± 2.021	10.533c ± 0.569	8.433d ± 0.493	7.133c ± 0.551	17.833c ± 1.258	8.633d ± 0.723	12.367c ± 1.436
	RA%	-29.35	-63.16	-57.68	-82.10	-90.11	-88.94	-77.32	-91.08	-86.25
Lufenuron	SA	88.967a ± 3.402	96.733a ± 1.159	96.033a ± 3.564	53.967a ± 3.453	77.167b ± 0.907	65.333a ± 2.082	67.167b ± 2.743	83.367b ± 2.413	58.967a ± 1.168
	RA%	5.00	0.21	4.65	-8.27	-9.50	1.34	-14.58	-13.85	-34.43
Indoxacarb	SA	80.233b ± 4.455	54.867c ± 2.511	61.700c ± 2.524	13.767bc ± 1.3657	5.233d ± 0.681	4.333c ± 0.666	21.267c ± 2.369	7.433d ± 0.208	8.933c ± 0.971
	RA%	-5.31	-43.16	-32.76	-76.60	-93.86	-93.28	-72.95	-92.32	-90.07

- Data are expressed as the means ± standard deviations (STDEVs).

- Values given are the means of three replicates.

- Means with the same letter in each column are not significantly different (*P* < 0.05).

* Conc. = concentration ** SA= specific activity ***RA%= relative activity percentage.

### Trehalase activity

The trehalase activity in the control group remained high, reaching 64.4 µg glucose/min/g.b.w by day 5. The chlorantraniliprole and indoxacarb treatments markedly suppressed trehalase activity. By day 5, the enzyme levels decreased significantly to 7.1 and 4.3 µg glucose/min/g.b.w, respectively. Emamectin benzoate also significantly reduced trehalase activity, with values ranging from 18.0 to 21.7 µg glucose/min/g.b.w during the experimental period. In contrast, lufenuron had a minimal effect on trehalase activity, maintaining enzyme levels relatively close to those of the control group (53.9–65.3 µg glucose/min/g.b.w) (Table 3).

### Amylase activity

The amylase activity in the control group remained high, reaching 89.9 µg glucose/min/g.b.w by day 5. The chlorantraniliprole and indoxacarb treatments significantly reduced amylase activity. By day 5, the enzyme levels had declined to 12.4 and 8.9 µg glucose/min/g.b.w, respectively. Emamectin benzoate induced initial suppression of amylase activity (21.8 µg glucose/min/g.b.w on day 1), followed by a gradual increase over time, reaching 29.967 µg glucose/min/g.b.w by day 5. Lufenuron caused moderate reductions in amylase activity but maintained the highest enzyme levels among the treated groups throughout the experimental period (58.9–83.3 µg glucose/min/g.b.w) (Table 3).

### Transaminase and alkaline phosphatase enzymes

AST activity in the control group gradually decreased from day 1 to day 5, reflecting normal physiological variation. Among all the treatments, chlorantraniliprole induced the greatest increase in AST activity, particularly on day 1 (372.7 U/g) and day 3 (309.7 U/g). Indoxacarb also significantly increased AST levels throughout the experimental period, peaking on day 3 (325.7 U/g). Emamectin benzoate caused moderate but statistically significant changes in AST activity (253.3–341.3 U/g), which was lower than that of the chlorantraniliprole and indoxacarb treatments. Lufenuron resulted in AST values comparable to those of the control group, with only slight fluctuations. ALT activity remained high in the control group. Among the tested compounds, chlorantraniliprole and indoxacarb had the strongest effects on ALT activity. Emamectin benzoate moderately suppressed ALT, whereas lufenuron maintained ALT levels close to those of the control group (Table 4).

**Table 4:** Changes in transaminase enzyme activities in the 4^th^ larval instars of *S. littoralis* treated with the LC_25s_ of the tested compounds.

Treatment		AST			ALT			ALP		
		1 day	3 days	5 days	1 day	3 days	5 days	1 day	3 days	5 days
Control	Conc.*	349.333bc ± 3.055	285.667b ± 9.292	264.332bc ± 9.292	78.333a ± 3.786	56.167a ± 3.403	48.333a ± 2.082	945.333b ± 50.302	520.667b ± 14.012	418.333b ± 23.629
Emamectin benzoate	SA**	341.333c ± 3.215	272.333b ± 4.163	253.332c ± 1.528	78.667a ± 3.055	43.033b ± 1.002	41.367b ± 1.206	751.333c ± 31.021	333.333d ± 13.868	358.333c ± 13.013
	RA%***	-2.29	-4.67	-4.16	0.43	-23.38	-14.41	-20.52	-35.98	-14.34
Chlorantraniliprole	SA	372.667a ± 6.429	309.667a ± 5.686	271.667b ± 4.726	47.333c ± 3.215	24.833c ± 1.258	21.067c ± 1.677	361.333d ± 10.263	240.667e ± 9.866	164.333d ± 13.577
	RA%	6.68	8.40	2.77	-39.57	-55.79	-56.41	-61.78	-53.78	-60.72
Lufenuron	SA	345.333bc ± 3.055	277.333b ± 6.429	267.333b ± 2.082	71.333ab ± 3.215	48.533b ± 2.214	47.167a ± 1.258	1617.667a ± 56.536	700.667a ± 11.015	629.667a ± 22.502
	RA%	-1.15	-2.92	1.14	-8.94	-13.59	-2.41	71.12	34.57	50.52
Indoxacarb	SA	354.667b ± 5.508	325.667a ± 9.452	294.333a ± 4.041	66.333b ± 3.215	22.833c ± 2.940	18.967c ± 0.681	894.667b ± 20.793	378.667c ± 7.095	416.333b ± 5.686
	RA%	1.53	14.00	11.35	-15.32	-59.35	-60.76	-5.36	-27.27	-0.48

- Data are expressed as the means ± standard deviations (STDEVs).

- Values given are the means of three replicates.

- Means with the same letter in each column are not significantly different (*P* < 0.05).

* Conc. = concentration ** SA= specific activity ***RA%= relative activity percentage.

Alkaline phosphatase (ALP) activity in the control group remained at moderate levels, showing a gradual decline over time, which was consistent with normal physiological variation. Lufenuron treatment resulted in the most pronounced increase in ALP activity, reaching 1617.7 mU/g.b.w on day 1 and remaining elevated throughout the experimental period. In contrast, chlorantraniliprole consistently suppressed ALP activity, decreasing to 164.3 mU/g.b.w by day 5. Indoxacarb and emamectin benzoate also significantly reduced ALP activity, particularly from days 3–5, indicating delayed enzymatic suppression.

### Alpha esterases

**Fig. 2:**
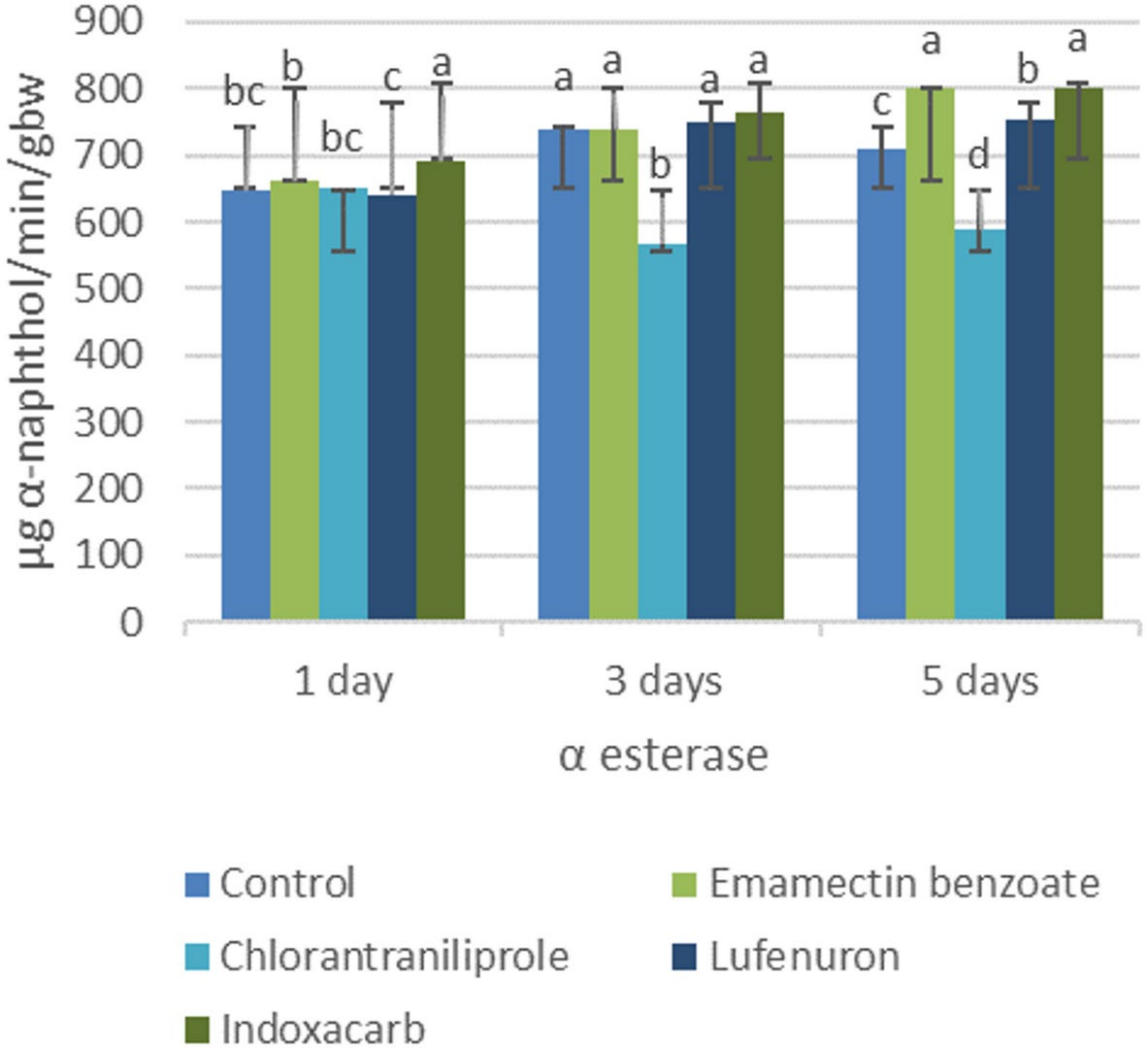
Biochemical changes in 4^th^ instar larvae of *S. littoralis* treated with the LC_25_ of the tested insecticides: α-esterase. The data are expressed as the means ± standard deviations (STDEVs). The values given are the means of three replicates. The values for each day marked by the same letters are not significantly different according to Tukey’s HSD test at *p* < 0.05. Those with different letters are significantly different.

Among the insecticides, the α-esterase activity of the treated larvae varied significantly. The indoxacarb and emamectin benzoate treatments resulted in marked increases in α-esterase activity, which peaked on day 5 (802.7 and 799.7 µg α-naphthol/min/g.b.w, respectively). In contrast, chlorantraniliprole caused a significant reduction in α-esterase activity from days 3–5 (566.3 and 588.7 µg α-naphthol/min/g.b.w, respectively). Lufenuron maintained α-esterase activity near control levels, which gradually increased over time (Fig. 2).

## Discussion

The use of a reference standard compound provides a reliable framework for evaluating and comparing insecticide efficacy. Therefore, both the toxicity index and potency level approaches were employed in the present study to ensure a robust comparative assessment. Compared with chlorantraniliprole, pyridalyl, indoxacarb, spinetoram, imidacloprid, chlorfenapyr, fipronil, novaluron, spinosad, and dinotefuran, the superior toxicity of emamectin benzoate confirms its greater effectiveness against cotton leafworm larvae–^19,22,36,37^. The observed variation in efficacy ranking between the LC_50_ and LC_90_ levels, particularly the lower performance of chlorantraniliprole at the LC_90_, may be attributed to its specific mode of action. Although chlorantraniliprole is recognized as a strong feeding deterrent^38^, its toxicological activity primarily targets the insect musculature, including the mandibular muscles essential for feeding, which may influence mortality patterns at relatively high lethal concentrations. Overall, the relatively similar slopes of the regression lines suggest homogeneous susceptibility within the tested larval population, supporting the reliability of the toxicity estimates obtained in this study.

The pronounced reduction in total protein content following exposure to chlorantraniliprole and indoxacarb suggests strong inhibition of protein synthesis and metabolic disruption. These effects may be attributed to feeding suppression, impaired digestion, and cellular degradation. In support of this interpretation, Gamil et al.^20^ reported that the total protein and carbohydrate contents were 29.23 and 7.55 mg/g.b.w., respectively, following LC_50_ exposure to indoxacarb in fourth instar larvae of *S. littoralis*. The decline observed with emamectin benzoate is consistent with its neurotoxic mode of action, which disrupts neuromuscular function and consequently reduces metabolic activity. Previous studies have similarly associated emamectin exposure with the inhibition of growth-related enzymes^39^, further explaining the observed protein depletion. Conversely, the negligible effect of lufenuron on total protein levels may be explained by its classification as an insect growth regulator (IGR). Lufenuron primarily interferes with chitin synthesis and cuticle formation rather than directly affecting protein metabolism, particularly during short-term exposure. The marked depletion of carbohydrate reserves following treatment with chlorantraniliprole and indoxacarb indicates substantial disruption of carbohydrate metabolism. This reduction may result from the inhibition of key metabolic pathways such as glycolysis or gluconeogenesis, in addition to feeding suppression and tissue damage. Consistent with these findings, Ammar et al.^40^. reported significant decreases in carbohydrate and protein contents in *S. littoralis* strains selected with indoxacarb compared with susceptible populations. The significant carbohydrate decline observed with emamectin benzoate further supports its role in inducing energy depletion. Its neurotoxic action likely causes neuromuscular paralysis, reducing feeding activity and consequently limiting energy intake, which accelerates the depletion of carbohydrate reserves. Conversely, the relatively stable carbohydrate levels recorded in lufenuron-treated larvae suggest that this compound does not acutely disrupt primary energy metabolism. As an insect growth regulator, lufenuron primarily interferes with molting and cuticle formation, exerting sublethal and developmental effects rather than directly impairing immediate metabolic pathways.

Invertase plays a critical role in carbohydrate metabolism by catalyzing the hydrolysis of sucrose into glucose and fructose, which are essential substrates for cellular respiration. Therefore, reductions in invertase activity reflect impairments in sucrose utilization and energy production. The marked suppression of invertase activity by chlorantraniliprole suggests strong inhibition of sucrose metabolism. This effect may be linked to its mechanism of action as a ryanodine receptor modulator, which induces muscular paralysis and subsequent metabolic disruption^41,42^. Similarly, the significant decrease observed with indoxacarb may be attributed to its sodium channel–blocking activity, which interferes with nerve impulse transmission, disrupts feeding behavior, and consequently reduces enzyme induction associated with carbohydrate metabolism. The moderate decline followed by partial recovery in emamectin benzoate–treated larvae may indicate some degree of physiological adaptation or comparatively lower direct interference with sucrose-metabolizing enzymes. Conversely, the negligible effect of lufenuron on invertase activity supports its classification as an insect growth regulator that primarily targets chitin synthesis and cuticle formation rather than directly affecting primary energy metabolism. Trehalase plays a fundamental role in insect physiology by hydrolyzing trehalose (the principal hemolymph sugar) into the glucose required for energy production and chitin biosynthesis^43,44^. Therefore, the suppression of trehalase activity directly reflects impaired carbohydrate mobilization and developmental processes. The extreme reduction observed with chlorantraniliprole and indoxacarb indicates severe disruption of energy regulation and carbohydrate metabolism. The markedly lower enzyme activities than those in the control samples suggest a profound metabolic imbalance that may ultimately lead to physiological collapse. The significant decrease recorded under emamectin benzoate treatment further supports its disruptive influence on glucose availability. Reduced trehalase activity likely limits glucose release, impairing the energy supply for growth and development, which may contribute to larval mortality. Conversely, the relatively stable trehalase activity in lufenuron-treated larvae aligns with its mode of action as a chitin synthesis inhibitor. Since its primary target is cuticle formation rather than direct interference with carbohydrate metabolism, its limited impact on trehalase activity is expected.

Amylase is a key hydrolytic enzyme responsible for starch digestion, supplying the glucose necessary for insect growth and development. Its enzymatic efficiency contributes to stress tolerance and metabolic stability^45^. The pronounced suppression of amylase activity by chlorantraniliprole and indoxacarb indicates severe impairment of starch metabolism. Reduced enzymatic breakdown of starch likely limits glucose availability, resulting in starvation-like conditions and developmental arrest. The transient suppression followed by partial recovery observed with emamectin benzoate may reflect delayed enzyme induction or compensatory physiological mechanisms aimed at restoring carbohydrate metabolism under toxic stress. In contrast, the relatively high amylase activity maintained in lufenuron-treated larvae supports its classification as a growth regulator with limited direct interference in primary digestive metabolism. Its primary effect on chitin synthesis explains its relatively moderate effect on starch hydrolysis. Transaminases such as AST and ALT are widely recognized as biochemical indicators of tissue integrity and metabolic stress. Elevations in these enzymes generally reflect cellular damage, membrane destabilization, or disruption of protein metabolism pathways. The marked increase in AST activity following chlorantraniliprole exposure suggests acute tissue stress, possibly associated with mitochondrial membrane destabilization resulting from its action as a ryanodine receptor modulator. Similarly, the significant AST elevation observed with indoxacarb indicates pronounced systemic toxicity, likely linked to its sodium channel–blocking mechanism and subsequent metabolic imbalance. The comparatively moderate alterations induced by emamectin benzoate indicate a lower cytotoxic profile relative to chlorantraniliprole and indoxacarb, although its neuromuscular blockade may still contribute to metabolic disturbance and reduced protein turnover. The limited changes observed under lufenuron treatment support its classification as a chitin synthesis inhibitor with minimal early-stage internal tissue disruption. The observed increases in AST and ALT activities in treated larvae are consistent with previous findings for several insect growth regulators and insecticides. Elevated transaminase activities have been reported following exposure to pyriproxyfen, flufenoxuron, and teflubenzuron^46^, and such enzymatic alterations are commonly interpreted as indicators of metabolic stress or insecticide-induced tissue damage. Furthermore, in vivo exposure to chlorantraniliprole and lufenuron has been shown to increase AST and ALT activities in a concentration- and time-dependent manner^47–50^, supporting the biochemical responses recorded in the present study.

Alkaline phosphatase is a key enzyme involved in membrane transport, nutrient absorption, and maintenance of cellular integrity, particularly in the midgut epithelium. Therefore, fluctuations in ALP activity serve as sensitive indicators of epithelial stress or tissue damage. The marked elevation observed following lufenuron treatment may reflect intestinal epithelial disruption and compensatory stress responses associated with tissue renewal or degeneration. As an insect growth regulator, lufenuron interferes with molting processes and cuticle formation, which may indirectly affect gut structure and function. In support of this interpretation, Ismail^13^ reported significant induction of ALP following exposure to lufenuron and emamectin benzoate. Conversely, the persistent suppression of ALP activity under chlorantraniliprole treatment suggests direct cellular toxicity or enzyme inhibition. Its neuromuscular mode of action may induce systemic stress, energy depletion, and impaired epithelial function, leading to reduced enzyme production or activity. The delayed reductions observed with indoxacarb and emamectin benzoate further indicate their progressive cytotoxic effects. Decreased ALP activity may correspond to impaired membrane transport and reduced nutrient absorption, likely secondary to feeding inhibition and metabolic disruption. Overall, these findings demonstrate that insecticides differ markedly in their impact on midgut enzymatic integrity, reflecting their distinct modes of action and physiological targets. α-Esterase is a crucial detoxification enzyme involved in hydrolyzing ester bonds and plays an important role in insecticide resistance and metabolic defense mechanisms. The significant increase in α-esterase activity following indoxacarb and emamectin benzoate exposure suggests that enzyme induction is part of a compensatory detoxification response. Increased esterase activity is commonly associated with increased metabolic processing of xenobiotics, reflecting the activation of defensive biochemical pathways under insecticidal stress. However, Abd El Aziz^51^ reported significant inhibition of α-esterase activity in fourth instar larvae of *S. littoralis* treated with indoxacarb and emamectin benzoate. This discrepancy may be attributed to differences in experimental conditions, exposure durations, or concentration levels, highlighting the complexity of enzymatic responses to insecticides. The significant suppression observed with chlorantraniliprole suggests either direct inhibition of detoxification pathways or cytotoxic damage impairing enzyme synthesis. Such inhibition may compromise the metabolic defense capacity of the larvae. The relatively stable esterase activity of lufenuron supports its classification as a chitin synthesis inhibitor with limited acute cytotoxicity. Its gradual enzymatic induction pattern indicates moderate metabolic adjustment rather than strong detoxification stress, which is consistent with its slower, growth-disruptive mode of action.

## Conclusion

Biochemical analyses clearly revealed that the tested insecticides had markedly different effects on the metabolic profile of *Spodoptera littoralis* larvae, reflecting their distinct modes of action. Emamectin benzoate exhibited the highest toxicity, whereas indoxacarb presented the lowest toxicity, as indicated by the LC_50_ values. Exposure to sublethal concentrations (LC_25_) resulted in significant reductions in total protein and carbohydrate contents, accompanied by pronounced alterations in key enzymatic activities related to carbohydrate metabolism (invertase, trehalase, and amylase), transamination processes (AST and ALT), phosphatase activity (ALP), and detoxification mechanisms (α-esterase). These biochemical disturbances were time-dependent and became more evident with increasing post-treatment duration. Overall, the results indicate that, in addition to their acute toxicity, the evaluated insecticides induce substantial physiological and metabolic disruptions in *S. littoralis* larvae. The generated data provide a robust scientific basis for the rational selection and judicious use of these insecticides within integrated pest management (IPM) programs, promoting effective control of the cotton leafworm while minimizing environmental risks and delaying the development of insecticide resistance.

## Data Availability

All the data generated or analyzed during this study are included in this published article and its supplementary information files.
